# Physiological Adaptation of *Chromochloris zofingiensis* in Three-Phased Cultivation Performed in a Pilot-Scale Photobioreactor

**DOI:** 10.3390/life15040648

**Published:** 2025-04-14

**Authors:** Richard Bleisch, Yob Ihadjadene, Agnese Torrisi, Thomas Walther, Gunnar Mühlstädt, Juliane Steingröwer, Stefan Streif, Felix Krujatz

**Affiliations:** 1 Institute of Natural Materials Technology, Technische Universität Dresden, 01069 Dresden, Germany; richard.bleisch@tu-dresden.de (R.B.); yob.ihadjadene@etit.tu-chemnitz.de (Y.I.); thomas_walther@tu-dresden.de (T.W.); juliane.steingroewer@tu-dresden.de (J.S.); 2 Chair of Automatic Control & System Dynamics, Technische Universität Chemnitz, 09126 Chemnitz, Germany; stefan.streif@etit.tu-chemnitz.de; 3 Department of Industrial Engineering DII, University of Padova, 35131 Padova, Italy; agnese.torrisi@unipd.it; 4 PUEVIT GmbH, 01109 Dresden, Germany; gm@puevit.com; 5 Fraunhofer Institute for Molecular Biology and Applied Ecology, Department of Bioresources, 35392 Giessen, Germany; 6 biotopa gGmbH—Center for Applied Aquaculture & Bioeconomy, 01454 Radeberg, Germany

**Keywords:** *Chromochloris zofingiensis*, astaxanthin, lipids, proteins, carotenoids, photobioreactor

## Abstract

*Chromochloris zofingiensis* is a green alga that serves as a valuable source of lipids, proteins, and carotenoids. Compared to well-studied microalgal carotenoid producers, *C. zofingiensis* offers several advantages, including high biomass, lipid and carotenoid productivity as well as less susceptibility to contaminations. *C. zofingiensis* can achieve growth rates up to four times higher than those of *H. pluvialis* under optimal phototrophic conditions. Although several studies have examined its cultivation and carotenogenesis under different tropic growth modes at laboratory scale, few have focused on pilot-scale systems. The goal of this study is to investigate the microalga’s physiological adaptation in a 200 L tubular photobioreactor during a three-phase semi-continuous cultivation strategy, particularly focusing on the changes in macromolecular and pigment composition. After an initial biomass accumulation phase, a two-phased stress phase was applied combining nutrient depletion (phase 1) and osmotic salt stress conditions (phase 2). Following this procedure, the cellular protein content dropped to 44.7% of its initial level, while the lipid content rose by up to 320%. Additionally, the astaxanthin concentration increased from 1.1 mg/g_DW_ to 4.9 mg/g_DW_ during the last osmotic stress phases, aligning with results from published laboratory-scale studies.

## 1. Introduction

Livestock farming represents the main source of protein to meet the needs of the world’s rapidly growing population [1]. However, concerns regarding the environmental impact of these production systems have driven the need to develop sustainable food sources [2]. Microalgae can play an important role in the production of sustainable food; moreover, they have a potential to contribute to the transition to a green, circular and carbon-neutral bioeconomy and provide health-promoting metabolites [3,4].

The nutritional value of microalgae has been reviewed extensively. The primary macromolecular compounds of interest for food applications are cellular proteins, lipids, carbohydrates and pigments [5,6]. Microalgal proteins are particularly noteworthy due to their higher quality and quantity in the biomass compared to other plant sources such as wheat, rice, or beans [6]. Moreover, their amino acid composition is comparable to that of some animal proteins, like egg proteins [7,8], making them an interesting candidate as a new source of protein on the way to a more plant-based healthy diet. The protein content of microalgal biomass varies depending on the species and cultivation conditions. Typically, unicellular eukaryotic microalgae such as *Chromochloris zofingiensis* reveal a protein content ranging from 40% to 60% of dry weight (DW), depending on abiotic and biotic cultivation conditions [9]. The lipids produced by marine microalgae provide a significant amount of valuable long-chain polyunsaturated fatty acids (PUFAs) such as docosahexaenoic acid (DHA) and eicosapentaenoic acid (EPA), which are renowned for their health-promoting properties [10]. In recent studies, it was shown that lipids produced by *C. zofingiensis*-related *Chlorella minutissima* can contain up to 39.9% long-chain omega-3 fatty acids, similar to animal sources like fish [11,12]. However, the lipid concentration of microalgae like *Nannochloropsis* sp. can reach up to 87% of the biomass DW, and that of *C. zofingiensis* can still exceed 65% [12,13,14]. Moreover, *C. zofingiensis* produces a variety of bioactive pigments, making it valuable for applications in pharmaceuticals, cosmetics, and food production additionally. These pigments include primary and secondary carotenoids with antioxidant properties, such as lutein, β-carotene, astaxanthin, canthaxanthin, and zeaxanthin [15]. Chlorophylls and phycobiliproteins (chromoproteins of cyanobacteria) exhibit additional antioxidant, anti-inflammatory, antitumor, and cardioprotective effects [16,17,18,19].

*C. zofingiensis*, formerly known as *Chlorella zofingiensis* and *Muriella zofingiensis*, is a green microalga classified under the class Chlorophyceae [20]. It undergoes a multi-phase cell cycle, starting with a growth phase where the cell volume increases under optimal conditions. In the second phase, *C. zofingiensis* performs multiple fission cycles, involving DNA replication and nuclear division [21]. Polynuclear cells of *C. zofingiensis* have been observed to produce up to 64 daughter cells (N = 2^n^), with the number of daughter cells determined by the number of DNA replication and nuclear division cycles before cell division. Once the parental cell wall ruptures, the daughter cells are released and enter the next multiple fission cycle. This adaptable microalga can be cultivated under various trophic conditions, such as phototrophic, heterotrophic, and mixotrophic modes. Different cultivation techniques stimulate the production of a wide range of macro- and micro-components, positioning *C. zofingiensis* as a promising candidate for biotechnological applications, including biofuel production, bioremediation, and the biosynthesis of high-value compounds [1,2].

*C. zofingiensis* offers several advantages in terms of biomass, lipid, and carotenoid productivity, with the potential to outperform traditional production hosts such as *Haematococcus pluvialis* [22,23]. *C. zofingiensis* achieves growth rates that are at least four times higher than those of *H. pluvialis*, with biomass productivity reaching up to 1.18 g/(L∙d) under optimal phototrophic conditions [23,24]. Several studies have reported its cultivation at the laboratory scale across different trophic growth modes [25,26,27]. However, limited experimental research has been conducted on the carotenogenesis and physiological adaptation of *C. zofingiensis* at pilot scale.

## 2. Materials and Methods

### 2.1. Microalgae Strain and Cultivation Conditions

The green microalga *C. zofingiensis* (SAG 211–14, Göttingen, Germany) was obtained from the Culture Collection of Algae at Göttingen University. The strain was maintained and pre-cultured in 250 mL shake flasks with a 50 mL working volume, cultivated at 25 °C, 150 rpm, and a 16:8 h light/dark photoperiod. Atmospheric CO_2_ served as the carbon source, and the culture was grown in Bristol’s Modified (BM) medium [28].

To explore the changes in the metabolic profile of *C. zofingiensis* during the growth phase and stress conditions (nutrient depletion and osmotic salt stress) in a pilot-scale 200 L photobioreactor (PBR), a three-phase semi-continuous cultivation strategy was implemented. The pre-culture was carried out in nine 150 mL cultivators (CellDEG GmbH, Berlin, Germany) in bubble-free aerated CO_2_-enriched air (2% *v*/*v*) at room temperature (22 ± 2 °C). The pilot-scale PBR cultivation was conducted in tubular PBRs (PUEVIT GmbH, Dresden, Germany) at 25 L and 200 L scales, under continuous illumination with photon flux densities of 100 µmol/(m^2^ s^1^) for the 25 L pre-culture and 200 µmol/(m^2^ s^1^) for the 200 L main culture, using the airlift principle (12 L/min, pressurized air without CO_2_ supplementation) at 22 ± 3 °C. An industrial growth medium composed of 0.3 g/L NPK fertilizer (Hauert MANNA Düngerwerke GmbH, Nürnberg, Germany; parts by weight: NaNO_3_: 3, P_2_O_5_: 15, K_2_O: 35, MgO: 5, Fe: 0.4) and tap water was used for the preculture and pilot-scale cultivation of *C. zofingiensis.* The initial pH of 6.8 rose during the biomass accumulation phase to a maximum of 7.8. The photon flux density was kept constant throughout the different cultivation and stress phases. Thus, no high photon flux intensities were applied for lipid and carotenoid accumulation, as is usually performed at laboratory scale to induce carotenogenesis.

**Figure 1:**
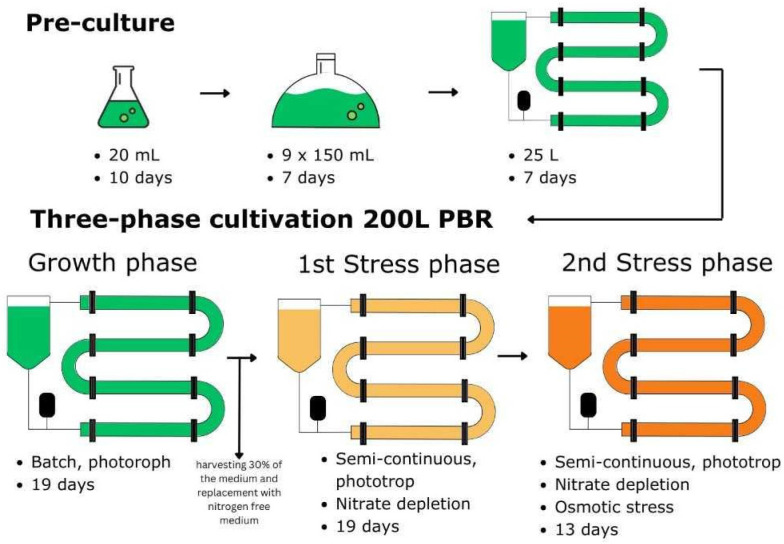
Workflow of the upscaling pre-culture process and the subsequent three-phased semi-continuous operation at the 200 L pilot-scale PBR.

Following the initial growth phase, which lasted 19 days, 30% of the culture was harvested and replaced with a nitrate-reduced medium to initiate the first nutrient depletion phase, decreasing the remaining nitrate concentration in the growth medium to approximately 10 mg/L. After an additional 19 days, the second phase was introduced, which involved nitrogen depletion combined with osmotic salt stress for 13 days, supplementing 17.5 g/L NaCl to the medium. During both phases, a semi-continuous cultivation approach was applied, with 20 L of culture being harvested and replaced with a nitrate-free medium ca. every 2 days, in order to collect enough sample volume for biomass analytics. The protocol was designed to gradually stress the biomass, enabling the observation of the algae’s transition from the green to the orange phase. A visualization of all the steps in the cultivation process is provided in Figure 1.

### 2.2. Analytics

The analytics used for this study are in accordance with, or slightly modified from, the protocols used in Bleisch et al. [29]. A brief description is provided in the following sub-chapters.

#### 2.2.1. Biomass Dry Weight (DW) Concentration

The biomass DW concentration was determined by filtering 10 mL of a microalgae sample through a pre-dried and pre-weighed (*W*_1_) glass microfiber filter with a 0.2 µm mesh size (Carl Roth GmbH KG, Karlsruhe, Germany). After filtration, filters containing biomass were washed with 20 mL deionized water; then, they were dried at 60 °C for 24 h in a laboratory heating cabinet (Salvis, Bender + Hobein, Zuerich, Switzerland) and weighed again (*W*_2_). The DW (g/L) was calculated using the following Equation (1):

(1) $$\mathrm{DW}\;[g/L]=\frac{{(W}_{2}-W_{1})}{10\;mL}\times 1000$$

The weight of the filter containing dried biomass (*W*_2_) and the filter without biomass (*W*_1_) was measured using a Kern 870 balance (Kern & Sohn GmbH, Balingen, Germany). Additionally, the optical density of the cultures was monitored at 750 nm using a GENESYS 150 UV/VIS spectrophotometer (Thermo Fisher, Waltham, MA, USA). Biomass samples were freeze-dried for macro-component analysis. The biomass was first separated from the medium by centrifugation (EBA 12, Andreas Hettich GmbH, Tuttlingen, Germany, 4 × 125 mL, 6 min, 5000 rpm) and washed once with phosphate-buffered saline (PBS, pH 7.2). The samples were then pre-frozen at −80 °C and freeze-dried in a second step (Beta 1–8 LSCbasic, Martin Christ Gefriertrocknungsanlagen GmbH, Osterode am Harz, Germany, −52 °C, <1 mbar, 5 days).

#### 2.2.2. Quantification of Nitrate

Nitrate samples were collected throughout the cultivation period. The supernatant was separated from the biomass by centrifugation at 5000 rpm for 6 min and then stored at −80 °C. Nitrate concentrations were measured using a modified protocol based on Witthohn et al. [30]. The modification involves the use of salicylic acid (≥99% purity) dissolved in ethanol (≥99% purity) in step 2 of the method, allowing for the detection of nitrate concentrations below 10 mg/L. Nitrate levels were quantified using the NanoQuant Infinite^®^ M200 PRO microplate reader (TECAN Trading AG, Männedorf, Switzerland) and calculated based on standard calibration curves.

#### 2.2.3. Pigments Extraction and Quantification

Chlorophylls a and b (*Chl* a, *Chl* b), total carotenoids (*Car*), and astaxanthin (*Atx*) were extracted using a modified method from Franke et al. [31]. Briefly, 1 mL of microalgal suspension (OD_750_: 0.7) was transferred into Eppendorf tubes and centrifuged to pellet the cells. After discarding the liquid phase, the pellets were resuspended in 1 mL dimethyl sulfoxide (DMSO), and then thermoshaken for 10 min at 70 °C and 700 rpm. The tubes were centrifuged again to separate the extracts from the remaining biomass, and the absorbance of the supernatant was measured at 480, 530, 649, and 665 nm. The quantification of *Chl* a, *Chl* b, and *Car* [mg/L] was performed using spectrophotometric analysis based on Wellburn’s method [32], employing the following Equations:

*Chl* a [mg/L] = 12.19*A*_665nm_ − 3.45*A*_649nm_ (2)

*Chl* b [mg/L] = 20.05*A*_649nm_ − 5.32*A*_665nm_ (3)

*Car* [mg/L] = (1000*A*_480nm_ − 2.14 *Chl* a − 70.16 *Chl* b)/220 (4)

where *A_λ_* is the absorbance of the extracts at wavelength *λ*. In accordance with the methods of Li et al. and Casella et al. [33,34], a calibration was performed using an analytical-grade astaxanthin standard (98%), to establish a relationship between its concentration and absorbance at 530 nm. The following Equation (5) was then used to calculate the astaxanthin (*Atx*) concentration:

*Atx* [mg/L] = (*A*_530nm_ + 0.0069)/0.1783 (5)

#### 2.2.4. Water and Ash Content

The water content of freeze-dried biomass samples was determined by sequential drying, with three cycles of 30 min each at 120 °C, using 1 g of dried biomass until a constant mass was reached (Kern 870, Kern & Sohn GmbH, Balingen, Germany). The ash content was determined gravimetrically (Kern 870, Kern & Sohn GmbH, Balingen, Germany) through ashing. The biomass was first pre-incinerated in a 100 mL crucible, and then incinerated in a muffle furnace (Controller B170, Nabertherm GmbH, Lilienthal, Germany) for 2 h at 550 °C.

#### 2.2.5. Elemental Analysis and Protein Content

The elemental nitrogen content was determined using the combustion elemental analysis (CHNS method). For this analysis, 20 mg of dried biomass was combusted in the presence of oxygen, resulting in the formation of various oxides. These oxides were subsequently analyzed to calculate the concentration of atomic nitrogen. The protein content was calculated using Equation (6), based on the nitrogen-to-total protein correlation factor established by Templeton and Laurens. They also demonstrated that the conversion factor is consistent for *Chlorella* species throughout the course of cultivation [35]. The results of Templeton and Laurens align with those of Sägesser et al. [36] and Safi et al. [37].

(6) $$\mathrm{Total}\;\mathrm{protein}\;[\%\;\mathrm{of DW}]={5.02\times c}_{N}\;\left[\%\;\mathrm{of}\;\mathrm{DW}\right]$$

#### 2.2.6. Total Lipid Content

Lipid extraction was carried out using the Soxhlet method [38], a lipophilic solvent-based extraction method that is widely used for processing microalgal biomass [14,25,39]. To ensure complete extraction, the biomass samples underwent acid hydrolysis. Two grams of freeze-dried biomass each sample were boiled in a 25% HCl solution for 2 h with constant stirring. The biomass was then neutralized by washing with 2 L of deionized water, filtered through a cellulose filter (ROTILABO^®^, Carl Roth GmbH & Co. KG, Karlsruhe, Germany, Type CR261, diameter: 47 mm), and dried at 60 °C for 24 h. Soxhlet extraction was performed using petroleum ether (40–60) in a SOXTHERM rapid extraction system (C. Gerhardt GmbH & Co. KG, Königswinter, Germany) for 80 min, with four extraction cycles at an extraction temperature of 150 °C. The lipid content was then determined gravimetrically from the extracted lipids.

## 3. Results and Discussion

### 3.1. State-of-the-Art of Chromochloris Zofingiensis Performance

As presented in Table 1, there are only a limited number of studies involving *C. zofingiensis* cultivations with larger than a 7.5 L culture volume, with even fewer reaching pilot-scale volumes exceeding 100 L. In general, the comparison of biomass, pigment and macro-component contents between studies is challenging due to the large number of experimental variables, such as cultivation system, cultivation scale, illumination conditions, media used or carotenoid induction like nutrient depletion, osmotic or light stress. To date, the largest reported photoautotrophic cultivation of *C. zofingiensis* is 240 L, although the culture in this study was just maintained in the green phase [40]. Moreover, the studies available to date have focused primarily on carotenoid biosynthesis under different trophic process modes in small-scale cultivation systems, highlighting the challenge to identify a cultivation and product induction strategy to realize both efficient carotenoid and lipid accumulation and scalability.

In this study, a 200 L tubular photobioreactor (PBR) was used to investigate the physiological adaptation of *C. zofingiensis* in a three-phase semi-continuous cultivation strategy focusing on the macro-molecular biochemical cell composition and pigment formation of *C. zofingiensis* at this pre-industrial scale.

### 3.2. Biomass Accumulation Phase

**Figure 2:**
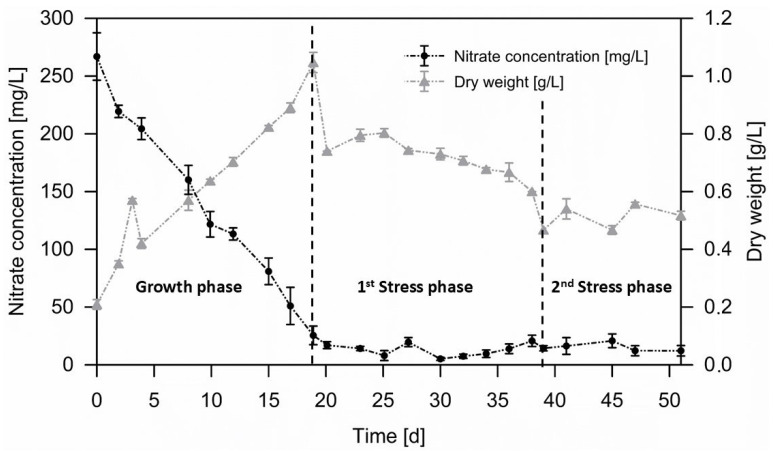
Biomass accumulation of *C. zofingiensis* and nitrate concentration (NO_3_^−^) during the three different cultivation phases, starting with an initial growth phase (day 0 to 19), a first nutrient-depletion stress phase (day 19–38) and a second stress phase combining nitrogen depletion and osmotic salt stress (day 39–51).

Figure 2 provides an overview on the biomass and nitrate development during the three-phased cultivation exploited for *C. zofingiensis* in this study. The initial nitrate concentration of 266.9 mg/L is metabolized effectively in a linear progression during the first 19 days of growth, reaching a final concentration of 10 mg/L and a yield coefficient of 3.16 g_DW_/g_nitrate_. Similar nitrate-to-biomass yields of 3.2 g_DW_/g_nitrate_ for *C. vulgaris* [44] and of 2.8 g_DW_/g_nitrate_ for *C. zofingiensis* [45] have been reported in previous studies. At the onset of the initial nutrient depletion phase, the nitrate concentration decreased to approximately 10 mg/L and remained at this level until the end of the cultivation process. This minimum nitrate concentration, allowing for the growth of *Chlorella* algae, is consistent with the findings of Ajala et al., who reported the assimilation of nitrate microalgae down to a lower limit ranging from 7.7 mg/L to 20.3 mg/L [44].

During the initial 19-day growth phase, the biomass DW concentration increased from 0.27 g/L to 1.05 g/L, resulting in a biomass productivity of 0.041 g/(L∙d), which is the same productivity range reported in the study of Wood et al. [14], who achieved a value of 0.03 g/(L∙d) in 65 L PBRs with CO_2_ injections operated in phototrophic mode as well. The slight difference in biomass productivity may be due to the variations in light regimes, as the authors in Wood et al. used a 16:8 h light/dark photoperiod, together with a maintained temperature between 20 and 22 °C, which, according to Vitali et al. [25], is below the optimum for *C. zofingiensis*. In contrast, and in the same study, Wood et al. [14] were able to achieve higher productivity, 0.56 g/(L∙d), in mixotrophic mode using 30 g/L of glucose as an organic carbon source. Vitali et al. [25] observed higher productivities of 0.057 g/(L∙d) and 0.07 g/(L∙d) in phototrophic and mixotrophic modes, respectively, as well as maximum biomass concentrations of 1.22 g/L after 20 days, comparable to the 1.05 g/L maximum biomass concentration achieved in this study after 19 days. However, this result was obtained by working on a much smaller scale of 12 L. It should be noted that the optimization of biomass productivity was not in the scope of this study. In a recent study, it was shown that the photoautotrophic productivity of *C. zofingiensis* can be improved by a factor of 5 by exploiting a model-based lighting profile during the growth phase [46].

Due to the regular harvesting of the medium and microalgal cells and its replacement with nitrate-depleted medium, no further increase in biomass concentration occurs during the following two cultivation phases. During the first nitrogen depletion phase, the biomass concentration decreased from 0.74 g/L to 0.6 g/L, and in the second osmotic stress phase, it further decreased to 0.52 g/L. The semi-continuous cultivation strategy, combined with nitrogen depletion, induces this loss of biomass, which is further intensified by the osmotic stress induced by salt addition, with biomass DW reductions of up to 21%. A comparable trend in biomass loss was observed in the study of Wood et al. [14].

Heterotrophic cultivation of *C. zofingiensis* has also been reported at scales up to 500 L (Table 1). While heterotrophic processes can achieve high biomass DW concentrations up to 200 g/L, there is ongoing debate regarding whether the additional investment in carbon sources, primarily glucose and sterile cultivation equipment, is justified. This is particularly relevant considering the reported yield coefficient of 0.36 g_DW_/g_glucose_ for the conversion of glucose into *C. zofingiensis* biomass [41] accompanied by lower product yields (see Section 3.2).

### 3.3. Pigments

**Figure 3:**
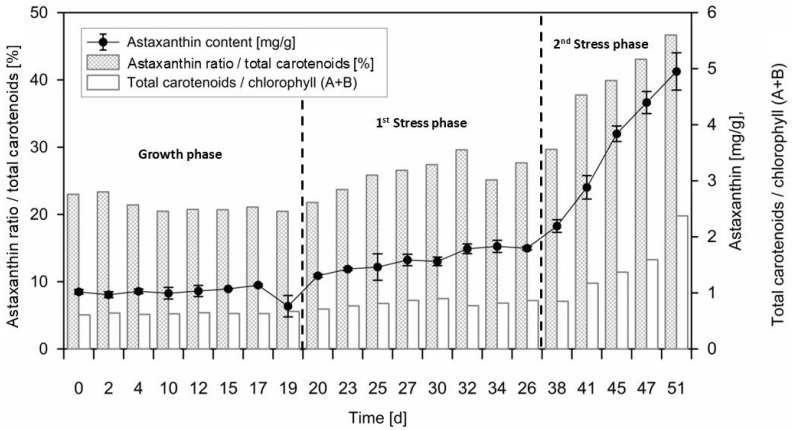
Pigment accumulation of *C. zofingiensis* during the different cultivation phases, starting with an initial growth phase (day 0 to 19), a first nutrient-depletion stress phase (day 19–38) and a second osmotic stress phase in combination with nutrient depletion (day 29–51). The values shown are of the astaxanthin concentration per dry weight biomass, the ratio of astaxanthin compared to the total amount of carotenoids in the biomass and the proportion of carotenoids to chlorophylls (stress response index). All proportionate values correspond to the corresponding values in mg/g.

**Figure 4:**
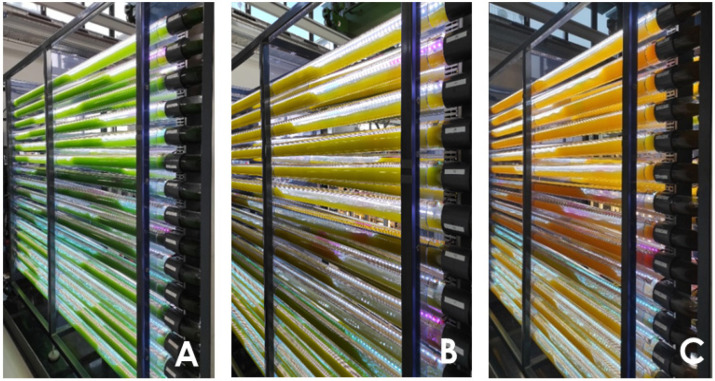
Pilot-scale 200 L photobioreactor during the three-phased photoautotrophic cultivation of *C. zofingiensis* during the three different cultivation phases, starting with (**A)** the initial growth phase (day 0 to 19), (**B**) the first nutrient depletion phase (day 19–38) and (**C**) the second phase combining nitrogen depletion and osmotic salt stress (day 39–51).

The changes in the cellular pigment composition of the *C. zofingiensis* biomass throughout the three-phased cultivation period are shown in Figure 3, along with photographs of the microalgae culture in the 200 L pilot-scale PBR in Figure 4 showing the visible adaption of the culture towards the changing environmental conditions. During the initial growth phase, the ratios of “carotenoids to total chlorophyll” and of “astaxanthin to total carotenoids” remained stable at 0.51 to 0.6, respectively. Throughout the initial 19 day growth phase, the astaxanthin content was at a low cellular level of ca. 1 mg/g, which was also reported in previous studies for physiological, growing “green” *C. zofingiensis* cells [41,45]. Following the initiation of nitrate depletion after 19 days, the ratio of carotenoids to total chlorophyll shifted toward the carotenoid fraction with a stress response index of 0.85 to 0.9. This stress response index was introduced by Sedjati et al. [47] to quantify the degree of adaptation of microalgae towards carotenoid accumulation and is equal to the ratio of total carotenoids and total chlorophyll concentration.

The cellular carotenoid concentration began to rise during the first nitrogen depletion phase at a rate of 0.195 mg/(g∙d). During the second stress phase combining the nutrient depletion with osmotic stress, the rate of carotenoid production increased significantly, reaching up to 0.883 mg/(g∙d). The cellular astaxanthin content also steadily increased during the first stress phase, from 1.05 mg/g to 2.2 mg/g, with an average rate of 0.075 mg/(g∙d). This trend continued during the second osmotic stress phase, which caused the culture to transition in color from green to yellow-brown and finally to reddish-orange (Figure 4). During this phase, the increase in astaxanthin content was significantly pronounced, rising by 0.41 mg/(g∙d) to a total of 4.9 mg/g after 51 days, yielding a stress response factor of 2.4, which is lower than that of 6.71 reported by Wood et al. [14] at lower scales. This suggests that the cellular stress level and carotenoid accumulation could potentially be further optimized; however, considering that this is the first study inducing carotenoid accumulation at an industrial-relevant scale. Similar rates of astaxanthin accumulation were observed in the study by Wood et al. for a 65 L PBR system using a two-stage process, ranging from 0.30 to 0.36 mg/(g∙d) [14]. The final astaxanthin concentration of 4.9 mg/g and the impact of osmotic stress are consistent with findings from various studies [22,48,49], which report astaxanthin concentrations of up to 6 mg/g under common stress factors such as high light intensities, osmotic stress, and nitrogen depletion at smaller scales. The cellular astaxanthin content of the gold standard of astaxanthin production, *Haematococcus pluvialis*, was reported to accumulate astaxanthin concentrations up to 25.92 mg/g [50] exposed to osmotic salt stress, a factor of 5 higher than that for *C. zofingiensis*. The application of novel chemical stimulants or high light exposure can further increase the cellular astaxanthin content by up to 46 mg/g [51]. However, the drawbacks of *H. pluvialis* cultivation related to high contamination risks and slow biomass productivity are also widely discussed [50], highlighting the need for novel carotenoid producers.

The transition from the green to the orange state of *C. zofingiensis* is induced by various stress factors, such as high light intensity and reduced nitrogen availability, which impair the photosystems and lead to the downregulation of chlorophyll. This process can be further enhanced by the addition of glucose [52,53]. While heterotrophic cultivation achieves high biomass DW concentrations, particularly at larger scales, the accumulation of astaxanthin per unit of biomass is typically lower compared to that in processes involving light induction, such as phototrophic and mixotrophic cultivation strategies, which was also demonstrated by other studies such as those by Wang et al. [27] and Minyuk et al. [45]. For instance, the maximum astaxanthin concentration of *C. zofingiensis* achieved in the cultivations by Chen et al. [41] was 1.44 mg/g, and that by Sun et al. [54] reached 1.02 mg/g.

### 3.4. Macroelements

**Figure 5:**
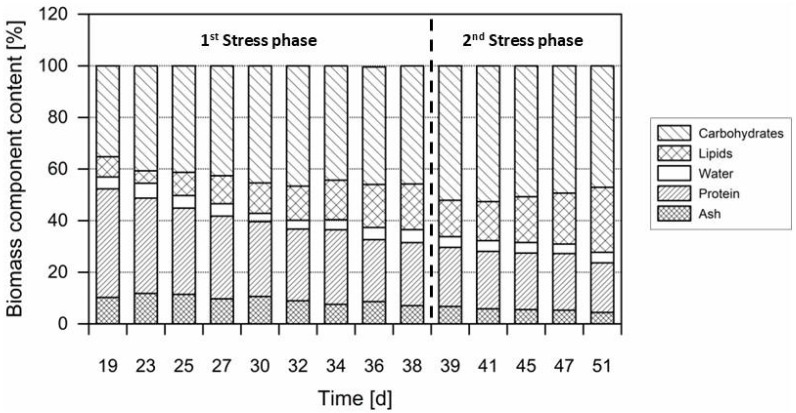
Cellular macroelements (biomass contents of carbohydrates, lipids, protein, ash and water) of *C. zofingiensis* during the two stress phases: the first nutrient depletion stress phase (day 19–38) and a second stress phase combining nitrogen depletion and osmotic salt stress (day 39–51) in a 200 L PBR.

While the macro-molecular composition of *C. zofingiensis* has been investigated in several studies, the effects of cellular stress inducers—such as pH fluctuations [13], nitrogen depletion [22,45], chemical stress [25], osmotic stress [25,41], and the impact of phytohormones and other stimulators [55]—have not been extensively reported in pilot-scale systems. Figure 5 presents the changes in the macromolecular biochemical composition of *C. zofingiensis* during the course of the 200 L PBR cultivation. Due to the high biomass requirement for analyzing the biochemical composition, the focus was on the nutrient depletion and osmotic stress (days 19–51) phase, where enough biomass was already available for the resource-intense laboratory analyses.

During the green growth phase, *C. zofingiensis* biomass accumulated up to 42.8% protein (day 19). With the onset of nutrient depletion, the protein content gradually declined until the end of cultivation, finally reaching 19.2%. Recent review articles have reported a comparable range of intracellular protein concentrations varying from 40.0% to 60.0% of the biomass DW in actively growing green biomass of *C. zofingiensis* [9] and approximately 15% in cells undergoing carotenoid accumulation [22].

Prior to nutrient depletion on day 19, the lipid content of *C. zofingiensis* was quantified at 7.8% of the DW. Upon exposure to the two stress conditions, the lipid content increased markedly, reaching 25.2% by the end of the second stress phase—more than three times higher compared to the initial level in non-stressed biomass. Under phototrophic conditions, Vitali et al. [25] reported a total lipid content of 30.3% of the biomass DW, consistent with the values observed in this study.

Several studies have evaluated the lipid productivity of *C. zofingiensis* under mixotrophic and heterotrophic conditions, reporting intracellular lipid quotas of approximately 42.0% and 62.0% of the biomass DW, respectively [14,22,23,25,41]. The presence of an additional carbon source significantly increases the intracellular carbon-to-nitrogen ratio, promoting the accumulation of storage compounds such as lipids. Attaining a comparable carbon input under autotrophic cultivation is generally not feasible.

Alongside lipid accumulation, carbohydrate content also rose by 16.7% of the biomass DW. A trade-off exists between carbohydrate and lipid synthesis, as both storage macromolecules share the same carbon precursors for biosynthesis. Starch synthesis typically precedes lipid formation [56,57], a pattern also observed in other green algae, as reported by Davey et al. [58]. Carbohydrates are thought to serve as an intermediate step in lipid biosynthesis, with starches being converted into fatty acids and subsequently into lipids. Moreover, it has been noted that under heterotrophic conditions, lipid synthesis is favored over carbohydrate accumulation, whereas mixotrophic conditions tend to promote carbohydrate formation over lipid storage [57]. Although lipids provide a more stable form of energy storage, they are less readily accessible than carbohydrates and are preferred by the industry due to their broad commercial applications.

In this study, the maximum carbohydrate content was measured at 52.7% of the biomass DW at the end of the nutrient depletion phase (day 38), which falls within the range of previously reported maximum carbohydrate concentrations of up to 66.9% of the biomass DW [23].

The water content of the biomass remained stable between 3.25% and 4.90% due to the standardized procedure of sample processing and drying. Residual water content is common in microalgal powders due to its highly hygroscopic properties [29]. Additionally, the ash content declined from 9.7% to 4.4% of the biomass DW during the course of cultivation. However, the ash content was found to be in the same range, as previously documented by Laurens and Wolfrum [59] (1.1% to 10.1%) and our previous findings [29].

## 4. Conclusions

The broad spectrum of bioactive compounds produced by *C. zofingiensis* positions it as a highly promising candidate for the food industry and the production of high-value metabolites such as lipids and carotenoids. This study demonstrated the feasibility of both phototrophic biomass production and stress induction at a pre-industrial scale of 200 L. The combined application of osmotic stress and nitrogen depletion significantly enhanced carotenoid synthesis, increasing total carotenoid content by a factor of 4.5 and astaxanthin accumulation by a factor of 2. These findings highlight the effectiveness of cultivation strategies that integrate multiple stress factors as a means of promoting carotenoid accumulation while circumventing the need for energy-intensive high-light treatments commonly employed in laboratory-scale studies, which are often unsuitable for large-scale application.

Moreover, *C. zofingiensis* proved to be a valuable protein source, particularly in its green growth phase, achieving a protein content of up to 42.8% of the biomass dry weight (DW). By modulating nutrient availability and salinity, the biochemical composition of *C. zofingiensis* can be strategically manipulated to favor either carotenoid and lipid accumulation or protein production.

Despite these advantages, the large-scale cultivation of *C. zofingiensis* still faces several challenges. Further optimization of biomass productivity is necessary to improve economic viability for food industry applications. Additionally, cost-effective downstream processing remains a critical factor that requires further investigation to facilitate the commercialization of *C. zofingiensis*-derived products.

## Figures and Tables

**Table 1:** Overview of *C. zofingiensis* cultivations at scales ranging from 7.5 to 500 L (considered pilot-scale), including phototrophic (po), mixotrophic (mix), and heterotrophic (ht) process operation documenting biomass dry weight concentration (DW), astaxanthin content (Astax) and the ratios of carotenoids to total chlorophyll (CAR/Chloro). All ht cultivations were carried out in a fermenter using glucose in varying concentrations as a carbon source. All po and mix cultivations employed different types of PBRs. Various culture stress factors, such as nitrogen depletion (Nx) and osmotic stress (oss), were applied to enhance lipid and carotenoid formation. Some cultivations followed a two-phase process with an initial growth and subsequent stress phase, with the durations combined as described, as well as different cultivations strategies like batch, fed batch (FBatch), or repeated Batch (repB) and the reactor system used: tubular PBR (Tub) vertical or horizontal, stirred reactor (SR), open pond (OP) or flat panel (FP).

Culture Conditions	DW [g/L]	Astax [%] of DW	CAR /Chloro	Total Macro Components [%] of DW				Source
				Lipids	Carbohydrates	Ash	Proteins	
Batch, Tub, po, 200 L, 19 d	1.05	0.10	0.66	10.2	35.2	7.8	42.1	this study
repB, Tub, po, Nx, oss, 19 d + 31 d	0.52	0.49	2.40	25.1	47.0	4.4	19.2	
Batch, SR, 500 L ht, 9 d	182.3	0.07	-	42.0	-	-	-	[41]
FBatch, SR, 7.5 L, ht, Nx, oss, 15 d	235.4	0.14	-	-	-	-	-	
Batch, Tub, po, 65 L, 15 d	-	-	0.23	11.7	25.0	9.9	34.8	[14]
Batch, Tub, mix, 65 L, 15 + 8 d	5.13	0.29	6.71	60.0	33.9	8.1	6.1	
Batch, FP, po, 240 L, 14 d	2.60	-	-	15.0	30.0	-	-	[40]
Batch, FP, po, 55 L, 14 d + 14 d, Nx	2.93	-	-	35.0	40.0	-	15.0	
Batch, Tub, 12 L, po, 20 d	-	-	-	20.0	-	-	-	[25]
Batch, Tub, 12 L, po, oss 20 d + 5 d	1.22	-	-	30.3	-	-	-	
Batch, SR, 20 L mix, 15 d + 5 d	121.5	0.56	-	-	-	-	-	[27]
Batch, OP, 100 L, po, 1 d	0.34	-	-	31.8	-	-	20.0	[42]
Batch, OP, 60 L, po, 9 d	0.9	-		54.5	-	-	-	[43]

## Data Availability

Data will be made available on request.
